# The plant-based by-product diets for the mass-rearing of *Acheta domesticus* and *Gryllus bimaculatus*

**DOI:** 10.1371/journal.pone.0218830

**Published:** 2019-06-27

**Authors:** Jaana M. Sorjonen, Anu Valtonen, Elina Hirvisalo, Maija Karhapää, Vilma J. Lehtovaara, Josefina Lindgren, Pertti Marnila, Patrick Mooney, Maarit Mäki, Hilkka Siljander-Rasi, Miika Tapio, Maria Tuiskula-Haavisto, Heikki Roininen

**Affiliations:** 1 Department of Environmental and Biological Sciences, University of Eastern Finland, Joensuu, Finland; 2 Natural Resources Institute Finland, Jokioinen, Finland; Universidad Nacional Autonoma de Mexico Instituto de Investigaciones en Ecosistemas y Sustentabilidad, MEXICO

## Abstract

Edible insect rearing could provide one alternative for protein production by having a smaller environmental impact than traditional livestock farming due to insects’ ability to convert organic side streams. Currently, the insect rearing industry utilizes soybeans as a major source of protein in the feeds. Protein-rich by-products of food industry could be used to replace them in insect feeds, but it is not known if they also meet the insects’ nutritional requirements. Our study evaluated the growth performance of two widely used edible cricket species, *Acheta domesticus* and *Gryllus bimaculatus* (Orthoptera: Gryllidae), on 18 experimental diets. The experimental diets included commercial chicken feeds and cricket diets, where soybean was partly and completely replaced with by-products from food industry: potato protein, barley mash, barley feed, compressed leftover of turnip rape and mix of broad bean and pea on three levels of protein. We found that the high- and medium-protein turnip rape and barley mash diets produced the highest yield and an increase in all performance variables. Overall, the high- and medium-protein diets produced the highest yield, growth and fastest development. Our results showed that by-products of food industry could be utilized as a part of the cricket feeds and thus advance the goals of circular economy.

## Introduction

The global food production system is under pressure to respond to the increasing demand for food in the future [1, 2]. The United Nations [3] estimates that by 2050, world population will increase to nine billion and demand for food by 70%. However, increase in global food production is severely challenged by land degradation, urban expansion, conversion of crops and cropland for non-food production, climate change, water scarcity, pest species infestations and overfishing of seas [4]. Agricultural production and how it changes land-use patterns are also major sources of greenhouse gas emissions with livestock production being one of the largest contributors [5]. In addition, the food industry creates a variety of by-product waste streams that are currently poorly utilized or left to decay [6]. Examples are bran, vegetable peelings, and residues of breweries that are often poorly utilized near the production areas. The current food production system requires a re-evaluation to provide a secure source of food for the growing populations in a more efficient and sustainable way.

Edible insects could provide an alternative for protein production and have a smaller environmental impact than traditional livestock [7, 8]. Due to their physiology, insects have the capacity to utilize resources, such as water and feed, more efficiently than traditional livestock. Therefore, insects have lower feed conversion rate (FCR) and better growth efficiency [9, 10]. Another environmental benefit of insects is their ability to convert organic side streams [9, 11, 12] indicating that many edible insect species have the potential to use a wide variety of plant material, such as food waste or by-products from agriculture and food industry [11]. This provides new interesting opportunities for sustainable insect rearing and the circular economy. The use of by-products as insect feeds could further lower the environmental impact of the insect rearing industry, but it should not reduce the insect performance, including developmental rate, relative growth rate, or their quality for human nutrition.

Due to their high content and beneficial composition of amino acids, soybeans have been commonly used as a major protein source in feeds of insect industry [13]. However, the use of soybeans has high environmental impact [14]. In order to improve sustainability of the insect rearing, it is necessary to find alternatives. These alternatives could come from side streams of food industry which could replace soybeans as a more sustainable protein source.

The goal of this study was to evaluate the growth performance of two widely-used edible insect species, house cricket *Acheta domesticus* (Linnaeus) and two-spotted cricket *Gryllus bimaculatus* (de Geer) (Orthoptera: Gryllidae), that were fed on feeds where soybean was replaced with plant-based by-products from food industry. These by-products were potato protein, barley mash, barley feed, turnip rape and a mix of broad bean and pea. We applied 18 experimental diets, which varied on their source and levels of protein. Our specific study questions were whether these diet treatments differ in 1) the yield, 2) the performance of crickets and 3) the efficiency of conversion of ingested food. The four performance variables were weight, relative growth rate, developmental rate and survival. In addition, the glycoalkaloid concentrations were measured from the *A*. *domesticus* crickets that consumed potato protein in their experimental diets.

## Material and methods

### Study insects

We used *A*. *domesticus* and *G*. *bimaculatus* crickets derived from laboratory populations at the University of Eastern Finland. We started the experiments with two-week-old nymphs when their weight accumulation is high. Before experiments the crickets had an equal mixed diet of two chicken feeds (Punaheltta Paras Poikanen and Puhaheltta Paras Kana, Suomen rehu, Finland) and reindeer feed (Poro-Elo 1; Suomen rehu, Hyvinkää, Finland) *ad libitum*. They were also offered water absorbed in tissue paper and pieces of fresh carrot.

### Experimental diets

The study included 18 diet treatments (Table 1) of which 14 of the experimental diets contained by-products (S1 Table). The four control diets included two chicken feeds and two types of modified Patton’s diet no. 16 [15]. These control diets have been widely used in cricket research and cricket rearing farms in Finland. The by-product diets were based on modified Patton’s diet no. 16 [15], where the soy-protein was replaced with protein from the by-products. The plant-based protein sources were chosen based on their availability as by-products of the Finnish food industry, and they comprised potato protein, barley mash, barley feed, turnip rape and mix of broad bean and pea (Table 1). The nutrient contents of each side stream diet (except potato protein) was repeated with the following three levels of protein: 30% in high (H), 22% in medium (M) and 15% in low (L). The potato protein diets were high in protein (30.5%) and they included two diets, one where half of the soybean was replaced with potato protein (potato-half), and the other where all of the soybean was replaced with potato protein (potato-all).

**Table 1:** The nutrient content and details of the 18 experimental diets.

	Protein (%)	Carbohydrate (%)	Fat (%)	Proportion of by-product in feed (%)	Source of by-product or control diet	Experimental round
***By-product diets***					Finnamyl Oy	
Potato-half ^a^	30.5	51.2	4.0	10		1
Potato-all ^a^	30.5	52.2	4.1	20		1
Barley mash-H ^b^	30.5	50.5	5.7	29	Honkavuori brewery Oy	2
Barley mash-M ^b^	22.5	58.0	6.5	41		2
Barley mash-L ^b^	15.0	66.0	5.4	20		2
Barley feed-H ^c^	30.0	51.0	5.2	15	Altia Oyj	2
Barley feed-M ^c^	22.5	58.2	7.4	44		2
Barley feed-L ^c^	15.0	66.0	6.5	31		2
Broad bean pea-H ^d^	30.0	50.4	3.8	30	Karita	3
Broad bean pea-M ^d^	22.5	58.2	3.9	30		3
Broad bean pea-L ^d^	15.0	66.0	4.2	13		3
Turnip rape-H ^e^	30.0	48.4	6.3	23	Kankaisten ljykasvit Oy	3
Turnip rape-M ^e^	22.5	56.8	5.9	5		3
Turnip rape-L ^e^	15.0	66.0	5.0	7		3
***Control diets***						
Chicken feed	15.2	56.6	4.4		Milka kanatäysrehu	1, 2, 3
Organic chicken feed	17.9	52.3	5.5		Luonnon Punaheltta	3
Patton’s modified diet no. 16	30.0	50.2	4.0		Patton, 1967	1
Patton+vitamins^f^	30.2	51.0	3.9			1

H = high-protein (30.5%), M = medium-protein (22.5%), L = low-protein (15.0%).

^a^By-product of potato flour production.

^b^By-product of beer production.

^c^By-product of ethanol production.

^d^Commonly used plant protein sources in Finland.

^e^By-product of rapeseed oil production.

^f^Vitamin mixture Vanderzant and salt mixture Wesson added.

The details of the diets were designed with WinOpti, a feed designing software program (AgroSoft WinOpti A/S, Agrosoft Ltd, Tørring, Denmark). In the feed designing program, we added information of all the ingredients used in the diets (S1 Table). Based on the nutritional values of ingredients, the program designs recipes for diets, where nutritional content, including protein, carbohydrate and fat levels can be set in advance. The diets were designed to reach one out of the three protein levels. When the protein level was lowered, the carbohydrate and fat levels were increased to replace protein. Due to the different nutritional values of the by-products, the proportion of the by-products in each diet was not constant (S2 Table).

### Experimental design

We conducted one experiment each for *A*. *domesticus* and *G*. *bimaculatus*. Each experiment was arranged as a randomized block design to determine the effect of diet on the yield, growth performance and the efficiency of conversion of ingested food (S1 Fig). The use of randomized block design controls the possible environmental variation and allows us to reveal the possible differences among treatments more efficiently. We used 3-litre plastic containers (11.0 x 18.5 x 18.5 cm), each having ventilation holes, 5 cm in diameter, covered with mesh in the lid. Ten cricket nymphs (15 days old) were randomized to each container. The experiments involved a total of 200 containers for each *A*. *domesticus* and *G*. *bimaculatus*.

The containers were randomly subjected to the experimental diets (Table 1), each with 10 replicates. The only exception was control diet chicken feed, which included 30 replicates. The diets were applied over the course of three experimental times (Table 1), with control diet chicken feed applied on each round. In each experimental time, the containers were randomized in blocks so that each block included two replicates of each experimental diet. The blocks represented thermally regulated growth chambers (70 x 125 x 50 cm) and there were five blocks altogether for each species and experimental time. Additionally, the blocks were located in two thermally regulated rearing rooms.

All containers were checked three times a week, and new feed was added to ensure insects were allowed to feed *ad libitum*. We provided nymphs with 20 ml of water absorbed in a paper tissue, 0.5–4.0 g of experimental diet, and ~3–4 g of fresh carrot to provide additional moisture and vitamins. The amount of experimental diet added was 0.5 g in the beginning of the experiment, but the amount was raised up to maximum of 4 g over the course of the experiment as the feed consumption of the crickets rose. In addition, the amount of leftover feed was weighed. During the first experimental time, the leftover feed was removed and recorded each time new feed was added. For the 2^nd^ and 3^rd^ experimental times the method was slightly changed and leftover feed was removed only when crickets reached adult stage and in the end of the experiment. The amount of offered feed was raised at the same time for all the containers in each experimental time. The feed was added always before it was fully consumed to allow insects to feed *ad libitum*. The leftovers of feces were not removed and the consumption of carrot was not taken into account in the total feed consumption. To provide hiding places and increase in surface area, two layers of egg carton (15 x 10 x 5 cm) were placed in each container. During the experiment, the containers were kept at 29°C ± 1.5°C and at 12L:12D photoperiod.

### Measured variables

The average weights of the nymphs in each experimental unit were measured at the beginning of the experiment. Over the course of the experiment, the fresh weight of leftover feed was recorded three times a week when containers were checked and feed was added. The weights of crickets were measured, and their sex was recorded individually when half of the crickets reached adult stage (adult weight) and one week after that (final weight). For each container, the experiment was terminated one week after half of the crickets reached adult stage.

For each individual, the relative growth rate (RGR) was calculated following Waldbauer [16]: fresh weight gain during feeding period (g) ∕ (duration of feeding (d) × mean fresh weight during the feeding period (g)). The weight gain was calculated using the following formula: adult weight − mean weight in the container at the beginning of the experiment. The mean fresh weight during the feeding period was calculated using formula: (mean start weight of individuals + adult weight of individual) ∕ 2. Duration of the feeding period was the days between the beginning of the experiment and when half of the crickets in each experimental unit reached adult stage.

For each container, we calculated four variables. Firstly, we calculated the yield of the crickets that is the sum of the weight of crickets alive at the end of the experiment. Secondly, we calculated the mean developmental rate using formula: 1 ∕ development time (d). The development time represented the days from the beginning of the experiment to when half of the crickets reached adulthood. Thirdly, we estimated the survival by calculating the number of individuals alive at the end of the experiment. Finally, we calculated the efficiency of conversion of ingested food (ECI) as: total weight gained (g) ∕ weight of ingested food (g) × 100 [16]. The weight of ingested food was calculated as: total weight of experimental feed added − total weight of experimental feed removed. Due to human error, in the first experimental time, there were missing values of leftover feed at certain time points. However, to calculate the total food consumption, some of the missing values were replaced with treatment means (60 values replaced out of 1 336).

### Glycoalkaloid analyses

Glycoalkaloid analyses were performed for *A*. *domesticus* crickets that had consumed the two potato protein diets. Only house crickets were chosen, as due to the similar physiology of the two cricket species, we assumed results of *A*. *domesticus* can inform us about the potential concentration of glycoalkaloids in both species. After the experiment, crickets were frozen and kept at −18°C. Four *A*. *domesticus* individuals from both treatments, including potato protein, were randomly selected for the glycoalkaloid analysis. The samples (cricket individuals) were freeze-dried (Alpha 1–4 LD Plus, Christ, Osterode am Harz, Germany: main drying of 22 h + final drying of 1 h). The glycoalkaloid analyses were carried out in Center of Food and Fermentation Technologies in Tallinn, Estonia. The UPLC-MS internal standard method was used to determine the α-solanine and α-chaconine concentrations. These results are shown in S3 Table.

### Statistical analyses

We fitted linear mixed models to ask, whether the diet treatment had an effect on the yield, developmental rate, weight and RGR of *A*. *domesticus* and *G*. *bimaculatus* (Table 2). For survival and ECI, we fitted generalized linear models (Table 2). The binary logistic model for events/trials data was used for survival, where the dependent variable was the number of individuals that survived in the end of the experiment (events) out of individuals in the start (trials). The ECI values did not meet normality; therefore, the gamma distribution model was used. The structure of all models is shown in Table 2. Firstly, the terms describing the structure of the experimental design (S2 Fig) were included as random factors. The male ratio and the start weight (measured from each container) were included as covariates. For some models it was not possible to include the experimental time as a random term (the model did not converge due to low number of experimental times) and therefore it was included in the model as a fixed term. If differences were found among diet treatments, Least Significant Difference (LSD) pairwise test was used to find out which treatments differed from each other. All statistical analyses were done with IBM SPSS Statistics 23.

**Table 2:** Details of statistical models.

	Yield^a^	Dev. rate^a^	Survival^a^	ECI^a^	Weight^b^	RGR^b^
***A*. *domesticus***	Linear Mixed Model	Linear Mixed Model	Generalized Linear Mixed Model, binary logistic model^c^	Generalized Linear Mixed Model^d^	Linear Mixed Model	Linear Mixed Model
Fixed terms	diet treatment	diet treatment, experimental time	diet treatment	diet treatment	diet treatment, sex, experimental time	diet treatment, sex, experimental time
Random terms	(experimental time*room), (experimental time*room*block)	(experimental time*room*block)	experimental time, (experimental time*room), (experimental time*room*block)	experimental time, (experimental time*room), (experimental time*room*block)	(experimental time*room), (experimental time*room*block), (experimental time*room*block*treatment*replicate)	(experimental time*room*block), (experimental time*room*block*treatment*replicate)
Covariates	male ratio, start weight	male ratio, start weight	male ratio, start weight	male ratio, start weight	start weight	start weight
***G*. *bimaculatus***	Linear Mixed Model	Linear Mixed Model	Generalized Linear Mixed Model^d^	Generalized Linear Mixed Model^d^	Linear Mixed Model	Linear Mixed Model
Fixed terms	diet treatment	diet treatment	diet treatment		diet treatment, sex	diet treatment, sex
Random terms	(experimental time*room*block)	experimental time, (experimental time*room), (experimental time*room*block)	experimental time, (experimental time*room), (experimental time*room*block)	experimental time, (experimental time*room), (experimental time*room*block)	experimental time, (experimental time*room), (experimental time*room*block), (experimental time*room*block*treatment*replicate)	experimental time, (experimental time*room), (experimental time*room*block), (experimental time*room*block*treatment*replicate)
Covariates	male ratio, start weight	male ratio, start weight	male ratio, start weight	male ratio, start weight	start weight	start weight

*Asterisks indicate the combinations of experimental time, room and block which were included as random effects to control for the dependency structure inherent in the experimental design.

^a^Measured from each container.

^b^Measured for each individual.

^c^Dependent variable was specified as the number of individuals that survived in the end of the experiment (events) out of individuals in the start (trials).

^d^Gamma distribution.

## Results

### Yield

**Fig 1:**
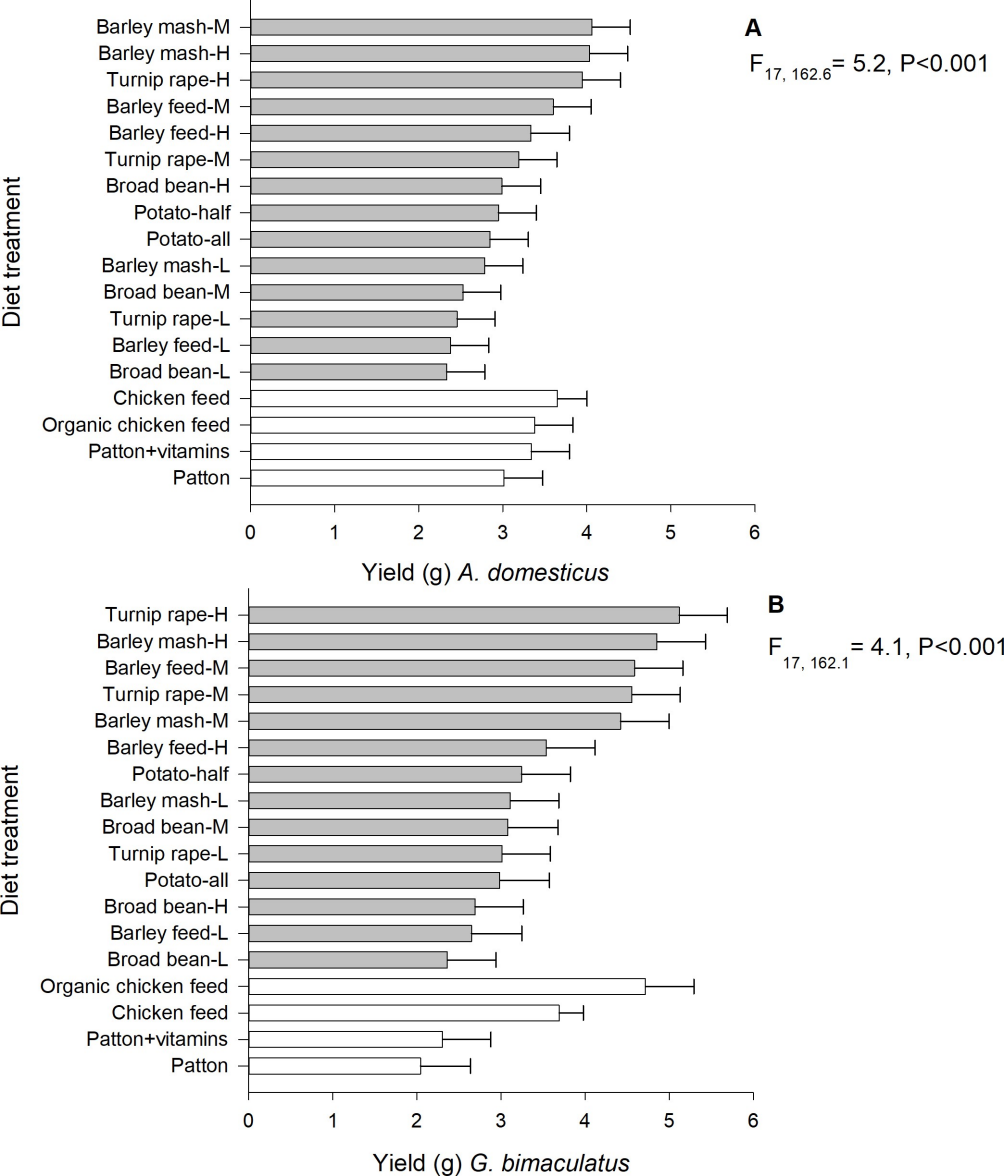
**Mean yield (g) per 10 reared individuals of *A*. *domesticus* (A) and *G*. *bimaculatus* (B) reared on experimental diets.** H = high-protein (30.5%), M = medium-protein (22.5%), L = low-protein (15.0%).

The yield of *A*. *domesticus* (linear mixed model; F_17, 162.6_ = 5.2, P < 0.001, n = 197) and *G*. *bimaculatus* (linear mixed model; F_17, 162.1_ = 4.1, P < 0.001, n = 196) differed significantly among the 18 experimental diet treatments. For *A*. *domesticus*, the highest yield was observed in medium- (estimated marginal mean: 4.10 ± SE 0.45 g) and high-protein (4.00 ± 0.45 g) barley mash diets (Fig 1A). In contrast, the lowest yield was observed in low-protein broad bean (2.30 ± 0.45 g) and low-protein barley feed (2.40 ± 0.44 g) diets. The start weight (P = 0.35) and experimental round (P = 0.68) did not explain the yield of *A*. *domesticus*, but the male ratio was negatively associated with it (parameter estimate = −1.4 ± SE 0.3, F_1, 171.8_ = 21.0, P < 0.001). For *G*. *bimaculatus*, the highest yield was observed in high-protein turnip rape (5.12 ± SE 0.57 g), high-protein barley mash (4.90 ± 0.58 g) diets and organic chicken feed (4.70 ± 0.57 g) (Fig 1B). The lowest yield was observed in Patton’s diet (2.00 ± 0.59 g) and Patton’s diet with added vitamins (2.30 ± 0.57 g). The start weight (P = 0.69), the male ratio (P = 0.26) or experimental round (P = 0.11) did not explain the yield of *G*. *bimaculatus*.

### Weight

**Fig 2:**
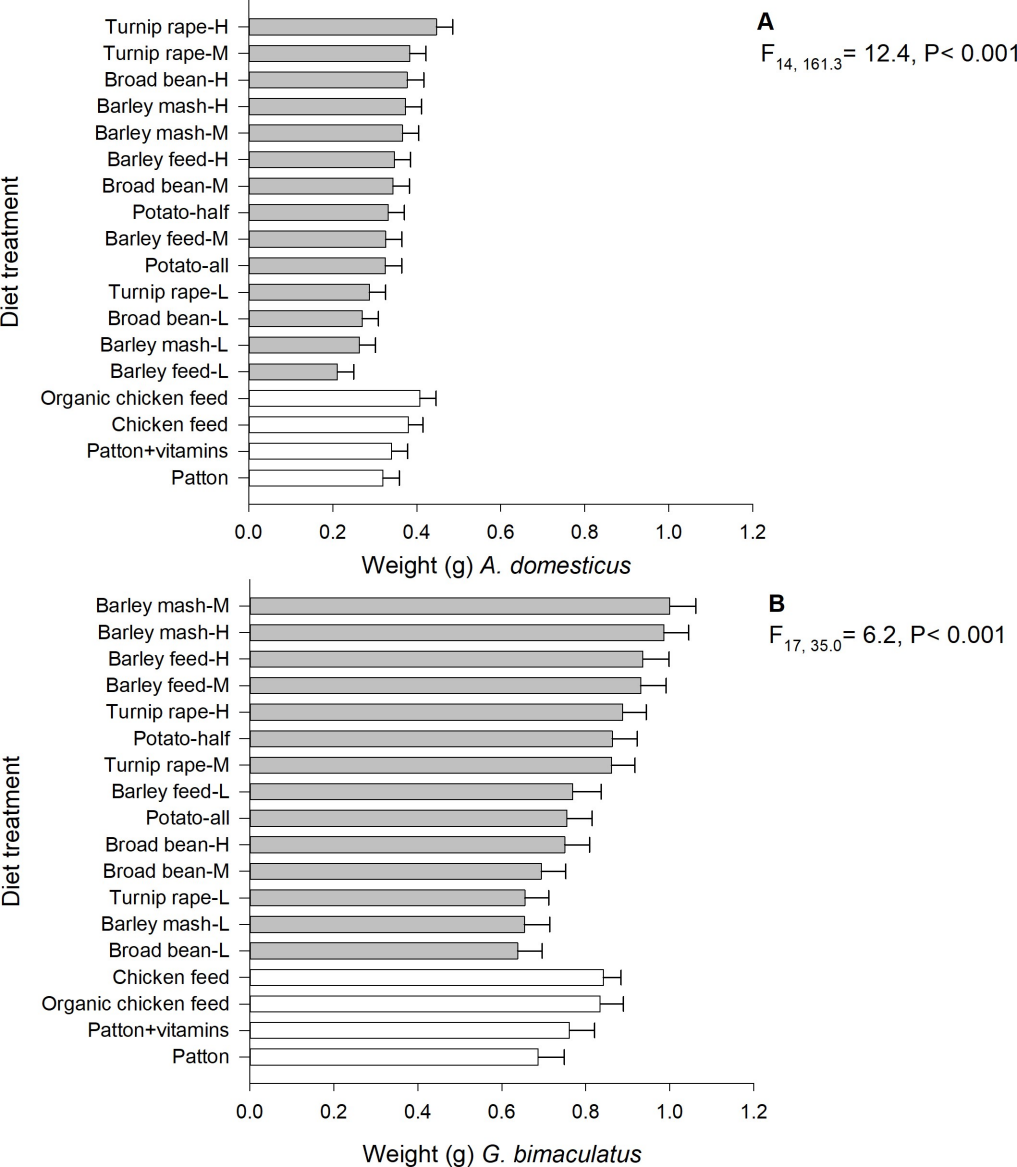
**Mean individual final weight (g) of *A*. *domesticus* (A) and *G*. *bimaculatus* (B) reared on experimental diets.** H = high-protein (30.5%), M = medium-proteins (22.5%), L = low-protein (15.0%).

The final weight (one week after half of the crickets reached adult stage) of both *A*. *domesticus* (linear mixed model; F_17, 161.3_ = 12.4, P < 0.001, n = 1547) and *G*. *bimaculatus* (linear mixed model; F_17, 35.0_ = 6.2, P < 0.001, n = 885) significantly differed among the 18 experimental diet treatments. However, the highest final weight was observed on different by-product diets (Fig 2). For *A*. *domesticus*, it was observed on high-protein turnip rape (marginal mean: 0.447 ± SE 0.039 g) and organic chicken feed (0.407 ± 0.039 g), while for the *G*. *bimaculatus*, it was on medium- (1.000 ± 0.061 g) and high-protein barley mash (0.986 ± 0.059 g). For both species, low-protein turnip rape, barley mash and broad bean-pea produced particularly low-weight individuals (Fig 2). The lightest *A*. *domesticus* individuals were observed in low-protein barley feed diet (0.221 ± 0.039 g), which is less than half of the weight produced by high-protein turnip rape diet (0.447 ± 0.039 g). The final weight of *A*. *domesticus* (F_2, 1473.2_ = 348.8, P < 0.001) and *G*. *bimaculatus* (F_2, 796.22_ = 205.6, P < 0.001) differed between sexes. The female crickets were heavier in both species compared to the male. The marginal mean weight of *A*. *domesticus* females and males was 0.459 ± 0.030 and 0.342 ± 0.030 g, respectively, while for *G*. *bimaculatus* females and males, it was 0.912 ± 0.028 and 0.626 ± 0.028 g, respectively. The start weight (P = 0.25) or experimental round (P = 0.90) did not explain the final weight of *A*. *domesticus*. Also, the start weight did not explain the final weight of *G*. *bimaculatus* (P = 0.24).

### The relative growth rate (RGR)

**Fig 3:**
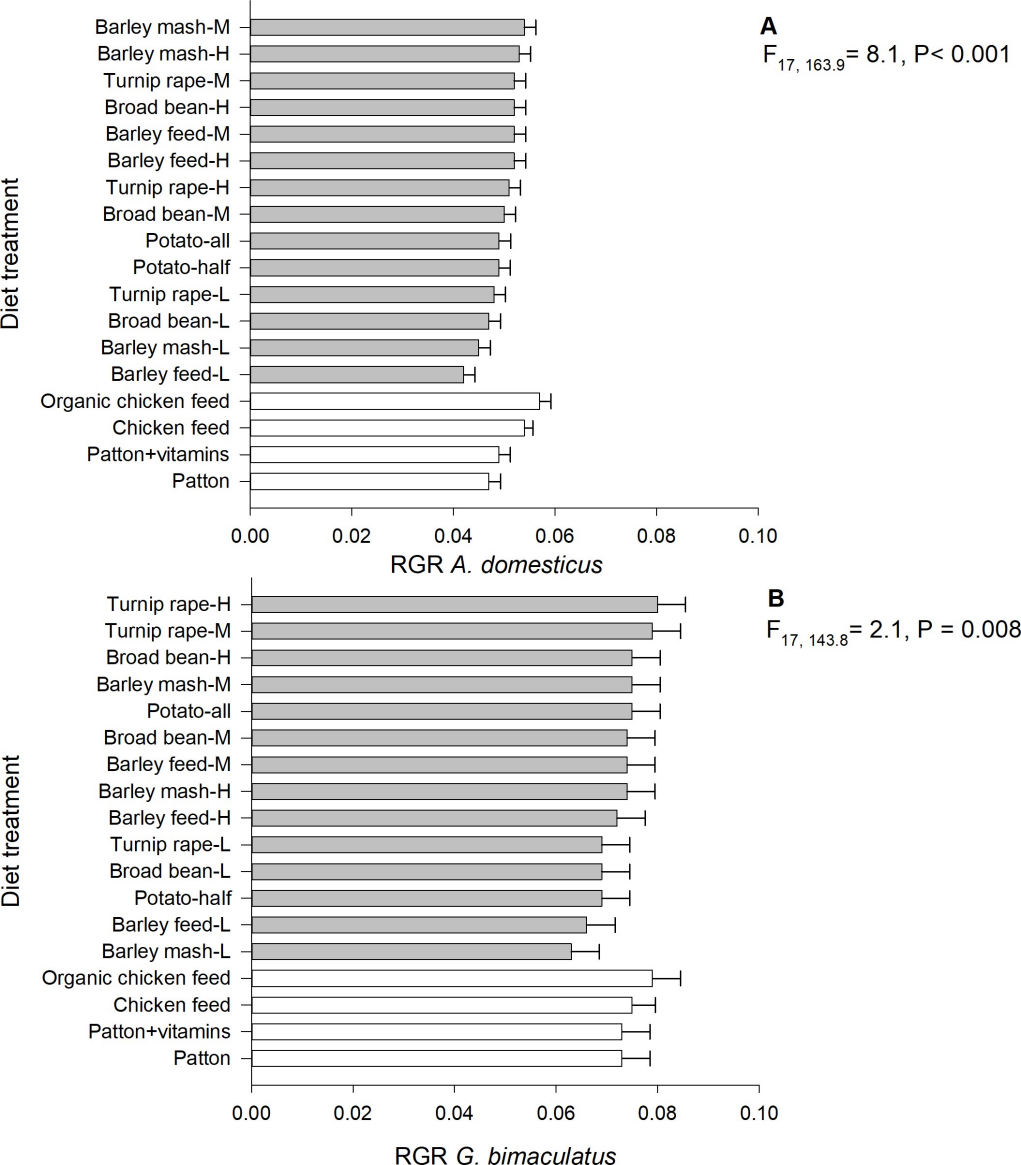
**Mean relative growth rate of *A*. *domesticus* (A) and *G*. *bimaculatus* (B) reared on experimental diets.** H = high-protein (30.5%), M = medium-protein (22.5%), L = low-protein (15.0%).

The RGR of *A*. *domesticus* differed significantly among the 18 experimental diet treatments (linear mixed model; F_17, 163.9_ = 8.1, P < 0.001, n = 1551). For *A*. *domesticus*, the highest RGR was observed in crickets fed on the organic chicken feed (marginal mean: 0.057 ± SE 0.002) and medium-protein barley mash (0.054 ± 0.002) (Fig 3A). The slowest RGR was found in individuals fed on low-protein barley feed (0.042 ± 0.002) and low-protein barley mash (0.045 ± 0.002). The experimental round (P = 0.89) did not explain the RGR of *A*. *domesticus*, but the start weight (parameter estimate = −0.20 ± SE 0.07, F _1, 168.2_ = 6.6, P = 0.011) and sex (F _2, 1354.6_ = 52.2, P < 0.001) did. Additionally, the RGR of *G*. *bimaculatus* differed significantly among the 18 diet treatments (linear mixed model; F_17, 143.8_ = 2.1, P = 0.008, n = 1009). For *G*. *bimaculatus*, the highest RGR was observed in high- (0.080 ± SE 0.005) and medium-protein turnip rape (0.079 ± 0.005) (Fig 3B). The slowest RGR was found in individuals fed on low-protein barley mash (0.063 ± 0.005) and low-protein barley feed (0.066 ± 0.006). The start weight (P = 0.21) did not explain the RGR of *G*. *bimaculatus*, but the sex did (F _2, 840.0_ = 25.9, P < 0.001).

### Developmental rate

**Fig 4:**
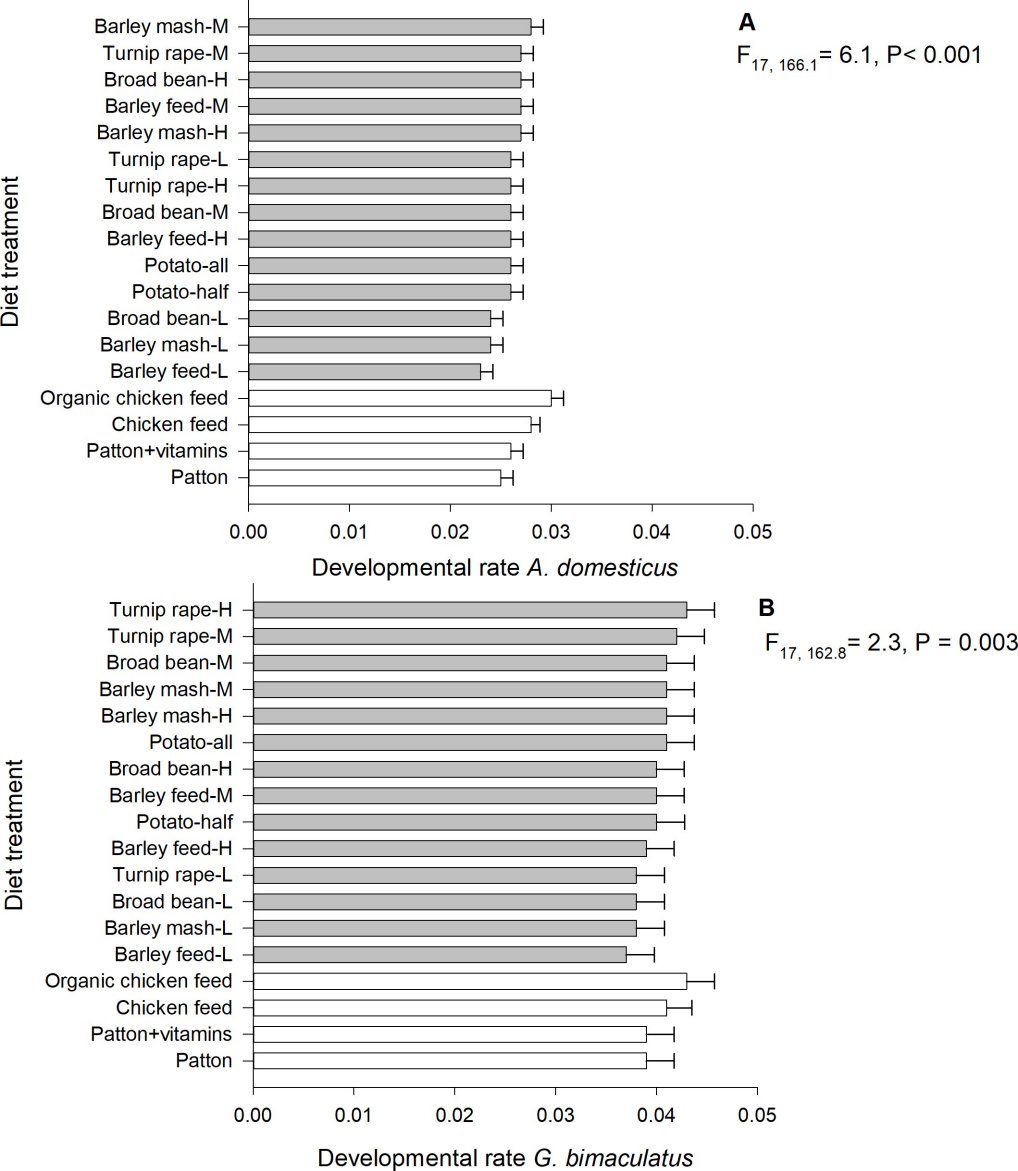
**Mean developmental rate of *A*. *domesticus* (A) and *G*. *bimaculatus* (B) reared on experimental diet.** H = high-protein (30.5%), M = medium-protein (22.5%), L = low-protein (15.0%).

The development time of *A*. *domesticus* and *G*. *bimaculatus* ranged between 34–45 and 24–28 days, respectively. The developmental rate (1/development time) of both *A*. *domesticus* (linear mixed model; F_17, 166.1_ = 6.1, P < 0.001, N = 200) and *G*. *bimaculatus* (F_17, 162.8_ = 2.3, P = 0.003, n = 198) differed significantly among the 18 diet treatments (Fig 4). For both cricket species, the fastest development was observed with organic chicken feed, on which *A*. *domesticus* individuals developed from 15-days-old nymphs to adulthood in 34 days (± SE 2 days) and *G*. *bimaculatus* in 24 days (± 2 days). Among the by-product diets, the fastest developmental rate was observed with medium-protein barley mash (marginal mean: 0.028 ± SE 0.001) and medium-protein turnip rape (0.027 ± 0.001) diets in *A*. *domesticus*, and high- (0.043 ± 0.003) and medium-protein turnip rape (0.042 ± 0.003) diets in *G*. *bimaculatus*. The slowest development was observed in low-protein barley feed diet for both cricket species, on which *A*. *domesticus* individuals developed to adulthood in 45 days (± 2 days) and *G*. *bimaculatus* in 28 days (± 2 days). The start weight (P = 0.91) or experimental round (P = 0.94) did not explain the developmental rate of *A*. *domesticus*, but the start weight was positively associated with the developmental rate of *G*. *bimaculatus* (parameter estimate = 0.030 ± SE 0.009, F_1, 173.7_ = 10.0, P = 0.002). The male ratio was negatively associated with the developmental rate of *A*. d*omesticus* (parameter estimate = −0.003 ± SE 0.001, F_1, 167.1_ = 10.6, P = 0.001), but positively with the developmental rate of *G*. *bimaculatus* (parameter estimate = 0.003 ± 0.001, F_1, 164.8_ = 7.6, P = 0.006).

### Survival

**Fig 5:**
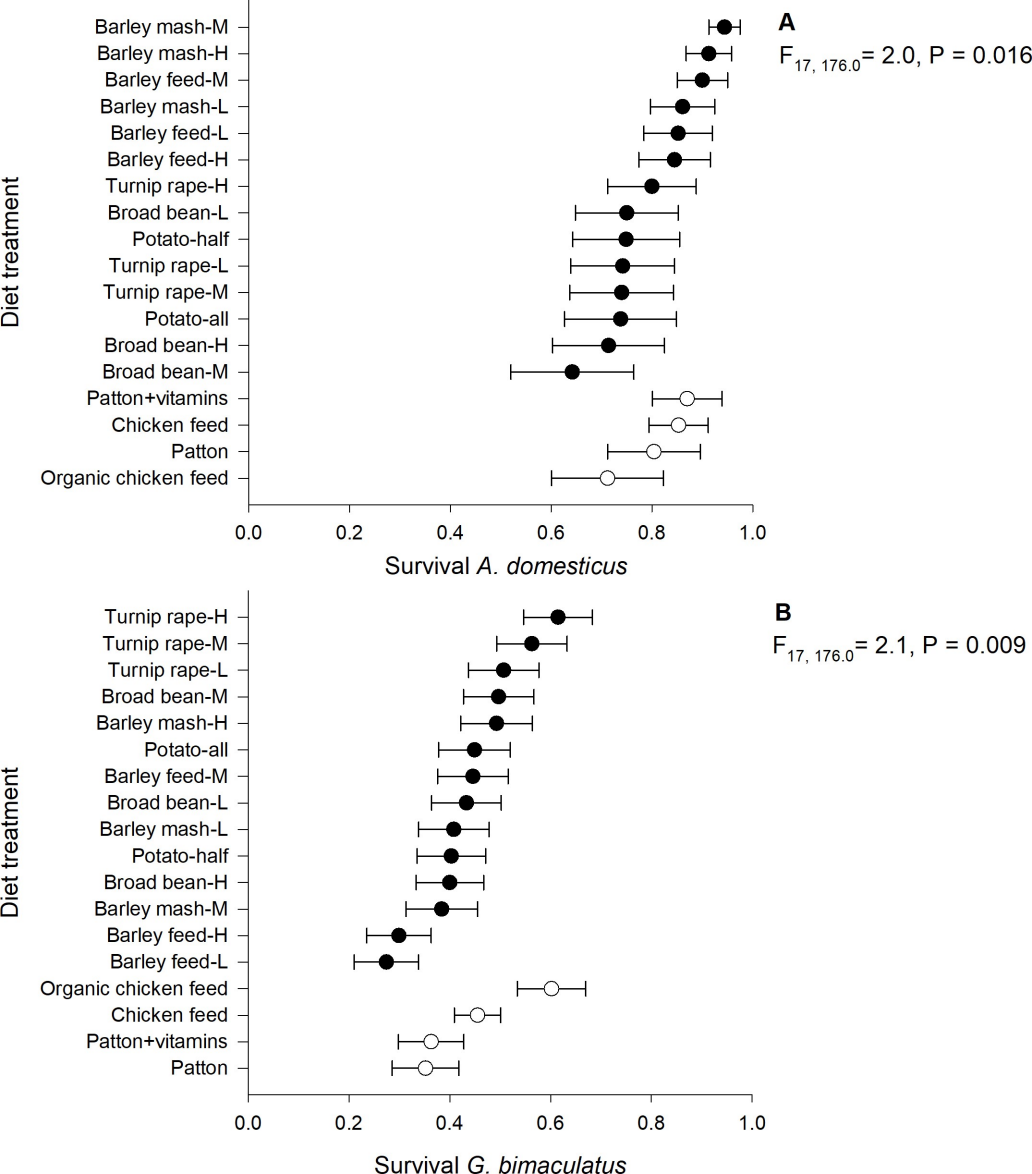
**Mean survival of *A*. *domesticus* (A) and *G*. *bimaculatus* (B) reared on experimental diets.** H = high-protein (30.5%), M = medium-protein (22.5%), L = low-protein (15.0%).

The overall survival of *A*. *domesticus* (80%) was approximately twice as high as the survival of *G*. *bimaculatus* (44%). The survival of *A*. *domesticus* (generalised linear mixed model; F_17, 176_ = 2.0, P = 0.016, n = 196) and *G*. *bimaculatus* (F_17, 176_ = 2.1, P = 0.009, n = 196) differed significantly among the 18 diet treatments. The highest survival was observed in *A*. *domesticus* with medium- (94.0 ± SE 3.1%) and high-protein barley mash (91.0 ± 4.5%), while in *G*. *bimaculatus*, it was with high-protein turnip rape (61.0 ± 6.8%) and organic chicken feed (60.0 ± 6.8%) (Fig 5). The start weight and male ratio did not explain the survival of *A*. *domesticus* (P = 0.92 and 0.07, respectively) and *G*. *bimaculatus* (P = 0.23 and 0.08, respectively).

### The efficiency of conversion of ingested food (ECI)

**Fig 6:**
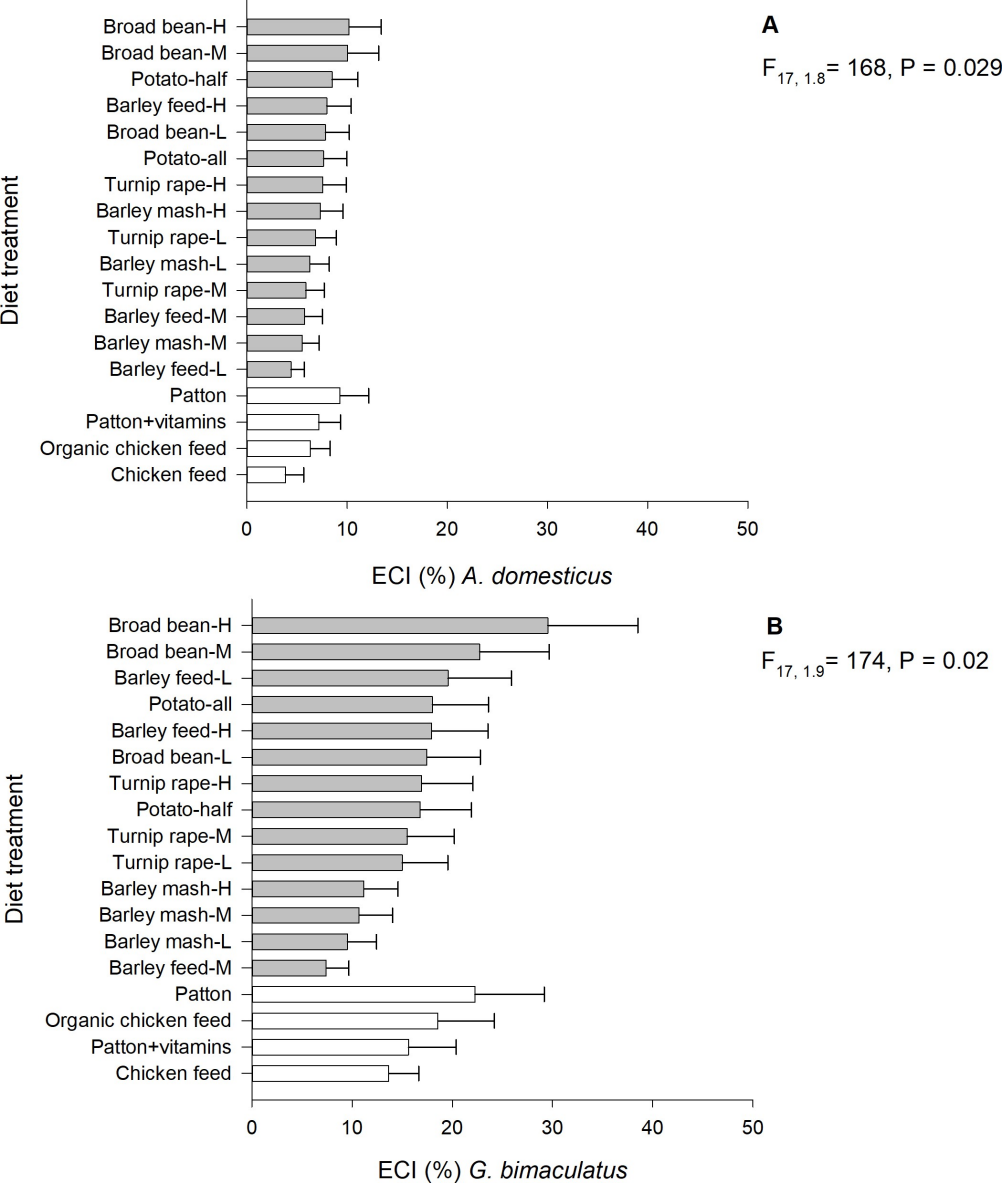
**Mean ECI of *A*. *domesticus* (A) and *G*. *bimaculatus* (B) reared on experimental diets.** H = high-protein (30.5%), M = medium-protein (22.5%), L = low-protein (15.0%).

The ECIs of *A*. *domesticus* (generalized linear mixed model; F_17, 1.8_ = 168, P = 0.029, n = 188) and *G*. *bimaculatus* (generalized linear mixed model; F_17, 1.9_ = 174, P = 0.02, n = 194) differed significantly among the 18 experimental diet treatments. The highest ECIs were observed in high- and medium-protein broad bean diets (*A*. *domesticus* 10.1 ± SE 3.2% and 10.0 ± 3.1%; *G*. *bimaculatus* 29.5 ± 9.0% and 22.7 ± 6.9%, respectively) (Fig 6). In contrast, the lowest ECI values for *A*. *domesticus* were observed in chicken feed (3.8 ± 1.8%) and low-protein barley feed diet (4.4 ± 1.3%). The male ratio (P = 0.07) did not explain the ECI of *A*. *domesticus*, but the start weight was negatively associated with it (parameter estimate = −5.0, F_1, 7.2_ = 168, P = 0.008). Additionally, the lowest ECI values for *G*. *bimaculatus* were observed in medium-protein barley feed diet (7.4 ± 2.3%) and low-protein barley mash diet (9.5 ± 2.3%). The start weight (P = 0.77) and male ratio (P = 0.14) did not explain the ECI of *G*. *bimaculatus*.

## Discussion

By-product protein sources have notable potential as feeds for *A*. *domesticus* and *G*. *bimaculatus*. These species accept, utilize and successfully develop with a range of plant-based by-product materials. Many of such diets produce enhanced growth, development and yield compared to the control diets. Previously, it has been shown that four edible insect species—house crickets (*A*. *domesticus*), Argentinean cockroaches (*Blaptica dubia* (Serville); Dictyoptera: Blaberidae), yellow mealworms (*Tenebrio molitor* (Linnaeus); Coleoptera: Tenebrionidae) and black soldier flies (*Hermetia illucens* (Linnaeus); Diptera: Stratiomyidae)—can be reared with diets that are composed of food manufacturing by-products [9]. In addition, Cambodian field crickets (*Teleogryllus testaceus* (Walker); Orthoptera: Gryllidae) has been successfully reared with by-products and weeds from food industry and agriculture [17]. However, diets composed mainly of organic waste or by-products (low value diets) may cause lower growth performance and survival of crickets [18, 19]. This suggests that diets including only by-products could lack important, nutritionally needed components for the development and growth of crickets. Our study, however, shows that by-products can be used as a protein source for crickets when the diet is in balance with other nutritional components, such as carbohydrates and fats.

The survival of crickets contributes more strongly to the yield than individual weights. The differences in the yield largely followed the same pattern as differences in survival among the diet treatments, but did not resemble much the patterns found in individual weights (S4 Table). The survival of *A*. *domesticus* was relatively high in by-product diets (64–94%) in comparison with previous studies. In our experiment, the highest survival rate in by-product diets was 94%, whereas survival rates of 6–80% in *A*. *domesticus* were previously reported [9, 20, 21]. In our study, high survival was expected because the by-product feeds were designed to meet the nutritional demand of crickets [13, 15], and our experiment was started with 15-days-old nymphs which might be more resistant than younger nymphs. However, the survival rates of *G*. *bimaculatus* were only half as high as in *A*. *domesticus*. The survival of *G*. *bimaculatus* in this study was comparable to that of *T*. *testaceus* reared with weeds and agricultural by-products (survival rates ranging between 15 and 40%) [17].

The different by-product diets cause variability within cricket species performance. In fact, the by-product protein sources that provided the best growth and developmental rate were different within the studied cricket species. The barley mash diets, medium and high in proteins, produced the fastest development in *A*. *domesticus*, while turnip rape produced the heaviest individuals. In contrast, the turnip rape, high or medium in proteins, produced the fastest development in *G*. *bimaculatus*, while the barley mash diets, high or medium in proteins, produced the heaviest individuals. In addition, the diet modified more strongly the developmental rate of *A*. *domesticus* compared to *G*. *bimaculatus*; the developmental time of *A*. *domesticus* ranged 11 days among the diet treatments (means of diets), while *G*. *bimaculatus* ranged only by 4 days. However, the developmental time of both cricket species was similar to previous reports in the literature, where it was for 4.5–11 weeks for *A*. *domesticus* [15, 20, 22], and 5 weeks [23] for *G*. *bimaculatus*. Although the focus of this study was to examine different by-products as a major protein sources in cricket’s feed, the slight differences among the carbohydrate and fat contents might have additionally modified the performance of crickets. The nutritional content of diets were designed as similar as possible with the feed designing program from available ingredients, but due to the practical constraints, there were slight differences in carbohydrate and fat content. In addition to protein content, carbohydrates and fats also play a role in growth and development [13, 24–26]. However, the medium and high protein turnip rape and barley mash diets seem to have a good balance of nutrients for growth of both of the studied cricket species. Overall, our results suggest that, in the future, different cricket species could be selected to utilize various by-products from the food industry, and that the diet can be used to modify the desired performance component specific to each species. Yet, the further analyses of the specific nutritional requirements of each of these species are still needed in the future.

The high protein content in diets was important for both studied cricket species revealing that the higher the protein level is in feed, the higher the yield and better the growth performance. Moreover, lower level of protein delays the development time and slows the individual biomass gain in insects in general [15, 25], suggesting that the diets with higher protein content are generally better for the growth and development of crickets. For example, Telang et al. [27] showed that the increasing dietary protein levels raised the storage protein levels of female tobacco budworm (*Heliothis virescens* (Fabricius); Noctuidae: Lepidoptera). The crickets are omnivorous insects thus their amino acid and protein requirement are relatively high relative to carbohydrates [26]. However, the studied crickets grew well with both medium (22.5%) and high levels (30.5%) of protein. This finding suggests that crickets could be reared with lower levels of protein than the previously reported optimum of 30% [15], if the feed meets the other nutritional requirements of crickets. Consequently, this lower amount of protein could decrease the costs of cricket feeds in the future.

Our results show that the high protein level in the by-product diets is associated with high efficiency of conversion of ingested food. Broad bean diet produced the highest ECI, which could be due to the most optimal amino acid composition of diet. Insect species typically require nine to ten essential amino acids for successful growth and development [13, 26]. The amino acid composition of the broad bean and pea is the most similar with soybean (S5 Table and S2 Fig). Feeds that contained lower levels of proteins and simpler amino acid compositions could modify the feeding behaviour of insects. To compensate the poorer nutrient content of food, insects can increase the consumption of lower quality food to fulfil nutritional requirements [25]. Furthermore, to regulate nutrient intake, insects have physiological mechanisms to adjust their feeding behaviour [28]. In general, the ECI was similar to other studies using by-products for house crickets [9, 10, 18]. However, the ECI values are not fully comparable to previous studies, because the crickets fed on the lower-value feeds might have consumed more carrot instead of the given feed. In addition, the weighed leftover feeds included remains of faeces, and the ECI values were calculated on fresh weight basis, making comparison to other studies difficult.

The potato glycoalkaloids did not exceptionally decrease the performance of the crickets even though the potato protein diets most likely contained glycoalkaloids. Our results showed (S3 Table) that the potato glycoalkaloids did not accumulate in the crickets, thus the glycoalkaloids should not cause a risk for human consumption. The glycoalkaloid concentrations of house crickets that consumed potato protein in the experimental diets were low; the means were 8.85 mg/kg (potato-half) and 7.65 mg/kg (potato-all). The highest allowed concentration of glycoalkaloids in raw potato for food in Finland is 200 mg/kg [29], meaning that crickets fed on potato protein are safe for human consumption. In addition, the lectin of broad bean [30] and the glucosinolates of turnip rape [31] have been shown to affect the performance and growth of insects. In this study, the house cricket survival was lower in the by-product feeds that contained these plant materials, but not significantly lower compared to control diets. Overall, the results suggest that the secondary metabolite residues in the plant materials used in this study appear to be too low to reduce the overall performance of crickets.

As our results indicate, there are many potential side stream-based protein sources that could be used in cricket feeds instead of soybean, thus enhancing circular economy, the efficient use of local resources and sustainable food production. Future studies should explore the best mix of different side streams as potential feeds and compare the sustainability of different alternatives (e.g. via Life Cycle Assessment [7]). In addition, the nutritional quality of insects is highly dependent on the given feed [9, 32, 33]. Thus further analysis of amino and fatty acids and chitin content would provide more diverse picture of insect’s quality for human food.

## Conclusions

This study shows that the by-products of food industry have notable potential as cricket feeds, since crickets accept, utilize and successfully develop with several plant-based side stream by-products. However, different by-product diets allowed the highest yield, fastest development and highest survival for each studied cricket species. The overall best by-product diet for *A*. *domesticus* was medium-protein barley mash, while for *G*. *bimaculatus*, it was high-protein turnip rape. Lastly, the use of side stream by-products could improve the environmental sustainability of insect feeds and insect rearing industry, and advance the targets of circular economy.

## Supporting information

S1 Table The detailed diet ingredients of the experimental diets. (DOCX)

S2 Table The nutritional content of ingredients (%). (DOCX)

S3 Table Mean α-solanine and α-chaconine content in *A. domesticus* crickets that consumed potato protein diets. (DOCX)

S4 Table The yield, performance and ECI of *A. domesticus* and *G. bimaculatus* on diet treatments (estimated marginal means ± standard error). (DOCX)

S5 Table Amino acid composition (%) of the major protein sources used in the experimental diets. The values are from the WinOpti–program that was used to design the feeds.
(DOCX)

S1 Fig Experimental design. For both species, we conducted experiments in three different times (experimental time). In each time, the experiment was conducted in two thermally regulated rearing rooms, and five blocks were located in these rooms. The block was a growth chamber, where the temperature was microregulated with heat cables. There were five blocks in total for each species during each experimental time. Each block included two replicates of each diet treatment in separate containers, each container having one treatment. The control diet chicken feed was applied in each experimental time. Each container had ten cricket individuals.
(PDF)

S2 Fig Amino acid composition (%) of the major protein sources used in the experimental diets. (TIF)
