# Supplementary material for: The plant-based by-product diets for the mass-rearing of Acheta domesticus and Gryllus bimaculatus
Source: PLoS One. 2019 Jun 27;14(6):e0218830. doi: 10.1371/journal.pone.0218830 (PMC6597079; doi:10.1371/journal.pone.0218830)
Supplement: S1 Table — (DOCX) [file pone.0218830.s001.docx]

**S1 Table. The detailed diet ingredients of the experimental diets.**

|  |  |  |  |  |  |  |  |  |  |  |  |  |  |  |  |  |  |  |
| --- | --- | --- | --- | --- | --- | --- | --- | --- | --- | --- | --- | --- | --- | --- | --- | --- | --- | --- |
| Ingredients (%) | Potato-half | Potato-all | Barley mash-H​ | Barley mash-M​ | Barley mash-L​ | Barley feed-H​ | Barley feed-M​ | Barley feed-L​ | Broad bean pea-H​ | Broad bean pea -M​ | Broad bean Pea-L​ | Turnip rape-H​ | Turnip rape-M​ | Turnip rape-L​ | Chicken feed^a^ | Organic chicken feed | Patton | Patton +vitamins |
| ***By-products*** |  |  |  |  |  |  |  |  |  |  |  |  |  |  |  |  |  |  |
| Potato protein | 10 | 20 |  |  |  |  |  |  |  |  |  |  |  |  |  | 0.5 |  |  |
| Barley mash |  |  | 28.7 | 41 | 20 |  |  |  |  |  |  |  |  |  |  |  |  |  |
| Barley feed |  |  |  |  |  | 15.67 | 43.9 | 31 |  |  |  |  |  |  |  |  |  |  |
| Pea |  |  |  |  |  |  |  |  | 15 | 15 | 6 |  |  |  |  |  |  |  |
| Broad bean |  |  |  |  |  |  |  |  | 15 | 15 | 7 |  |  |  |  |  |  |  |
| Turnip rape |  |  |  |  |  |  |  |  |  |  |  | 22.6 | 18 | 7.2 |  |  |  |  |
| ***Other ingredients*** |  |  |  |  |  |  |  |  |  |  |  |  |  |  |  |  |  |  |
| Corn gluten |  |  |  |  |  |  |  |  |  |  |  |  |  |  |  | 3.9 |  |  |
| Corn groat | 15 | 15 | 5 | 8 | 4 | 5 | 8 | 7 |  |  |  | 5 | 5 | 3 |  |  | 15 | 15 |
| Field mustard |  |  |  |  |  |  |  |  |  |  |  |  |  |  | x |  |  |  |
| Fish meal |  |  |  |  |  |  |  |  |  |  |  |  |  |  | x |  |  |  |
| Limestone |  |  |  |  |  |  |  |  |  |  |  |  |  |  | x |  |  |  |
| Milk powder | 7.1 | 7 | 12 | 8 | 4 | 12 | 8 | 5.1 | 8 | 5 | 3 | 8 | 5 | 3 |  |  | 7.2 | 7.1 |
| Oat |  |  |  |  |  |  |  |  |  |  |  |  |  |  | x | 2.7 |  |  |
| Plantoil | 2 | 2 | 2 | 2 | 2 | 2 | 2 | 2 | 2 | 2 | 2 | 2 | 2 | 2 | x |  | 2 | 2 |
| Powdered grass |  |  |  |  |  |  |  |  |  |  |  |  |  |  |  | 0.5 |  |  |
| Preliminary feed for chickens |  |  |  |  |  |  |  |  |  |  |  |  |  |  |  | 0.3 |  |  |
| Salts |  |  |  |  |  |  |  |  |  |  |  |  |  |  | x | 10 |  |  |
| Seaweed | 4 | 4 | 4 | 4 | 4 | 4 | 4 | 4 | 4 | 4 | 4 | 4 | 4 | 4 |  |  | 4 | 4 |
| Soybean | 17.6 |  | 24 | 8 |  | 28 | 10 |  | 22 | 7.9 |  | 20 | 4.8 |  | x | 18.3 | 38 | 40 |
| Vanderzant vitamin mixture |  |  |  |  |  |  |  |  |  |  |  |  |  |  |  |  |  | 2.5 |
| Wesson salt mixture |  |  |  |  |  |  |  |  |  |  |  |  |  |  |  |  |  | 2.5 |
| Wheat | 29.3 | 38.4 | 10 | 20.4 | 62 | 16.4 | 10 | 44.9 | 17.1 | 39.5 | 72 | 26.4 | 49.2 | 75.8 | x | 26 | 22 | 15.1 |
| Yeast (inactivated) | 15 | 13.7 | 14.3 | 8.6 | 4 | 16.9 | 14.1 | 6 | 17 | 12 | 6 | 12 | 12 | 5 |  |  | 11.8 | 11.8 |

^a^The ingredients of chicken feed marked as x since proportions not available.

H= High-protein level (30.5 %), M = medium (22.5 %), L = low (15%).
