# Supplementary material for: The plant-based by-product diets for the mass-rearing of Acheta domesticus and Gryllus bimaculatus
Source: PLoS One. 2019 Jun 27;14(6):e0218830. doi: 10.1371/journal.pone.0218830 (PMC6597079; doi:10.1371/journal.pone.0218830)
Supplement: S2 Table — (DOCX) [file pone.0218830.s002.docx]

**S2 Table. The nutritional content of ingredients (%).**

|  | Protein | Fat | Fiber |
| --- | --- | --- | --- |
| **By-products** |  |  |  |
| Potato protein | 80 | 2.5 | 0 |
| Barley mash | 21.16 | 8.19 | 15.64 |
| Barley feed | 14.7 | 9.81 | 16 |
| Pea | 19.78 | 0.95 | 4.9 |
| Broad bean | 25.8 | 1.29 | 6.88 |
| Turnip rape | 34.92 | 10.88 | 8.53 |
| **Other ingredients** |  |  |  |
| Corn groat | 10 | 1.3 | 0.8 |
| Milk powder | 35 | 1 | 0 |
| Plant oil | 0 | 100 | 0 |
| Seaweed | 7.5 | 3 | 7.5 |
| Soybean | 47.5 | 2.3 | 5 |
| Wheat | 11.07 | 2.24 | 2.21 |
| Yeast (inactivated) | 44 | 2 | 1.5 |
