# Supplementary material for: The plant-based by-product diets for the mass-rearing of Acheta domesticus and Gryllus bimaculatus
Source: PLoS One. 2019 Jun 27;14(6):e0218830. doi: 10.1371/journal.pone.0218830 (PMC6597079; doi:10.1371/journal.pone.0218830)
Supplement: S3 Table — (DOCX) [file pone.0218830.s003.docx]

**S3 Table. Mean α-solanine and α-chaconine content in *A. domesticus* crickets that consumed potato protein diets.**

| Treatment | α-solanine (mg/kg) | α-chaconine (mg/kg) | Total content (mg/kg) | n |
| --- | --- | --- | --- | --- |
| Potato-half | 4.225 | 4.625 | 8.850 | 4 |
| Potato-all | 3.975 | 3.650 | 7.625 | 4 |
