# Supplementary material for: The plant-based by-product diets for the mass-rearing of Acheta domesticus and Gryllus bimaculatus
Source: PLoS One. 2019 Jun 27;14(6):e0218830. doi: 10.1371/journal.pone.0218830 (PMC6597079; doi:10.1371/journal.pone.0218830)
Supplement: S4 Table — (DOCX) [file pone.0218830.s004.docx]

|  | Yield (g) | | Weight (g) | | Relative growth rate | | Developmental rate | | Survival | | ECI | |
| --- | --- | --- | --- | --- | --- | --- | --- | --- | --- | --- | --- | --- |
|  | *A. domesticus* | *G. bimaculatus* | *A. domesticus* | *G. bimaculatus* | *A. domesticus* | *G. bimaculatus* | *A. domesticus* | *G. bimaculatus* | *A. domesticus* | *G. bimaculatus* | *A. domesticus* | *G. bimaculatus* |
| Chicken feed | 3.650±0.353 | 3.692±0.287 | 0.380±0.027 | 0.842±0.042 | 0.054±0.002 | 0.075±0.005 | 0.028±0.001 | 0.041±0.002 | 0.853±0.059 | 0.455±0.046 | 3.84±1.823 | 13.64±2.993 |
| Organic Chicken feed | 3.017±0.452 | 4.719±0.575 | 0.407±0.032 | 0.834±0.056 | 0.057±0.002 | 0.079±0.005 | 0.030±0.001 | 0.043±0.003 | 0.712±0.111 | 0.602±0.068 | 6.33±1.953 | 18.55±5.644 |
| Patton's diet no 16 | 3.341±0.462 | 2.043±0.593 | 0.319±0.033 | 0.687±0.062 | 0.047±0.002 | 0.073±0.005 | 0.025±0.001 | 0.039±0.003 | 0.804±0.092 | 0.352±0.066 | 9.29±2.860 | 22.25±6.923 |
| Patton's +vitamins | 3.383±0.451 | 2.304±0.574 | 0.340±0.032 | 0.761±0.060 | 0.049±0.002 | 0.073±0.005 | 0.026±0.001 | 0.039±0.003 | 0.870±0.069 | 0.363±0.065 | 7.16±2.177 | 15.63±4.737 |
| Potato half | 2.948±0.451 | 3.244±0.581 | 0.332±0.032 | 0.863±0.059 | 0.049±0.002 | 0.069±0.005 | 0.026±0.001 | 0.040±0.003 | 0.749±0.106 | 0.403±0.068 | 8.50±2.584 | 16.78±5.125 |
| Potato all | 2.847±0.458 | 2.984±0.594 | 0.325±0.033 | 0.755±0.060 | 0.049±0.002 | 0.075±0.005 | 0.026±0.001 | 0.041±0.003 | 0.738±0.111 | 0.449±0.071 | 7.64±2.345 | 18.00±5.598 |
| Barley mash-H​ | 4.034±0.452 | 4.856±0.576 | 0.373±0.032 | 0.986±0.059 | 0.053±0.002 | 0.074±0.005 | 0.027±0.001 | 0.041±0.003 | 0.913±0.045 | 0.493±0.071 | 7.33±2.252 | 11.16±3.408 |
| Barley mash-M​ | 4.064±0.453 | 4.423±0.577 | 0.366±0.032 | 1.000±0.061 | 0.054±0.002 | 0.075±0.006 | 0.028±0.001 | 0.041±0.003 | 0.944±0.031 | 0.384±0.071 | 5.53±1.684 | 10.69±3.341 |
| Barley mash-L​ | 2.787±0.451 | 3.112±0.577 | 0.263±0.032 | 0.654±0.060 | 0.045±0.002 | 0.063±0.005 | 0.024±0.001 | 0.038±0.003 | 0.861±0.064 | 0.408±0.070 | 6.29±1.914 | 9.52±2.907 |
| Barley feed-H​ | 3.334±0.459 | 3.541±0.577 | 0.347±0.032 | 0.936±0.063 | 0.052±0.002 | 0.072±0.006 | 0.026±0.001 | 0.039±0.003 | 0.845±0.071 | 0.299±0.064 | 7.97±2.447 | 17.92±5.625 |
| Barley feed-M​ | 3.600±0.452 | 4.588±0.575 | 0.326±0.032 | 0.931±0.060 | 0.052±0.002 | 0.074±0.005 | 0.027±0.001 | 0.040±0.003 | 0.900±0.05 | 0.446±0.070 | 5.77±1.756 | 7.41±2.256 |
| Barley feed-L​ | 2.381±0.451 | 2.649±0.599 | 0.211±0.032 | 0.769±0.068 | 0.042±0.002 | 0.066±0.006 | 0.023±0.001 | 0.037±0.003 | 0.852±0.068 | 0.274±0.064 | 4.39±1.335 | 19.57±6.343 |
| Broad bean pea -H​ | 2.990±0.460 | 2.690±0.574 | 0.377±0.033 | 0.750±0.059 | 0.052±0.002 | 0.075±0.006 | 0.027±0.001 | 0.040±0.003 | 0.714±0.111 | 0.400±0.067 | 10.17±3.228 | 29.53±9.00 |
| Broad bean pea -M​ | 2.524±0.452 | 3.081±0.598 | 0.343±0.033 | 0.694±0.058 | 0.050±0.002 | 0.074±0.005 | 0.026±0.001 | 0.041±0.003 | 0.642±0.122 | 0.497±0.070 | 10.03±3.098 | 22.73±6.947 |
| Broad bean pea -L​ | 2.332±0.453 | 2.362±0.577 | 0.270±0.032 | 0.638±0.058 | 0.047±0.002 | 0.069±0.005 | 0.024±0.001 | 0.038±0.003 | 0.750±0.102 | 0.433±0.069 | 7.82±2.393 | 17.46±5.33 |
| Turnip rape -H​ | 3.948±0.452 | 5.12±0.573 | 0.447±0.032 | 0.888±0.056 | 0.051±0.002 | 0.080±0.005 | 0.026±0.001 | 0.043±0.003 | 0.800±0.088 | 0.615±0.068 | 7.59±2.317 | 16.92±5.14 |
| Turnip rape -M​ | 3.192±0.452 | 4.557±0.574 | 0.383±0.032 | 0.861±0.056 | 0.052±0.002 | 0.079±0.005 | 0.027±0.001 | 0.042±0.003 | 0.740±0.103 | 0.563±0.070 | 5.87±1.836 | 15.48±4.712 |
| Turnip rape -L​ | 2.455±0.452 | 3.012±0.574 | 0.287±0.032 | 0.655±0.057 | 0.048±0.002 | 0.069±0.005 | 0.026±0.001 | 0.038±0.003 | 0.742±0.103 | 0.507±0.070 | 6.84±2.089 | 14.99±4.559 |

**S4 Table.** **The yield, performance and ECI of A. domesticus and G. bimaculatus on diet treatments (estimated marginal means ± standard error).**
