# Supplementary material for: The plant-based by-product diets for the mass-rearing of Acheta domesticus and Gryllus bimaculatus
Source: PLoS One. 2019 Jun 27;14(6):e0218830. doi: 10.1371/journal.pone.0218830 (PMC6597079; doi:10.1371/journal.pone.0218830)
Supplement: S5 Table — The values are from the WinOpti–program that was used to design the feeds. (DOCX) [file pone.0218830.s005.docx]

**S5 Table. Amino acid composition (%) of the major protein sources used in the experimental diets.** The values are from the WinOpti –program that was used to design the feeds.

| Amino acid composition (%) | Soybean | Pea | Broad bean | Turnip rape- and rapeseed | Mash flour | Potato | Barley feed |
| --- | --- | --- | --- | --- | --- | --- | --- |
| Arginine | 7.36 | 8.42 | 10.32 | 6.42 | 4.75 | 5.44 | 4.29 |
| Phenylalanine | 5.11 | 4.95 | 4.76 | 4.54 | 5.28 | 5.06 | 5.34 |
| Histidine | 2.66 | 2.74 | 3.02 | 3.10 | 1.80 | 1.90 | 2.09 |
| Isoleucine | 4.91 | 4.11 | 4.18 | 4.32 | 5.49 | 4.43 | 4.29 |
| Leucine | 7.77 | 7.37 | 7.89 | 7.75 | 10.03 | 6.96 | 6.81 |
| Lysine | 6.13 | 7.47 | 6.84 | 6.42 | 3.70 | 6.33 | 3.66 |
| Methionine | 1.53 | 0.95 | 0.70 | 1.99 | 1.69 | 1.65 | 1.57 |
| Treonine | 4.09 | 3.79 | 3.60 | 4.87 | 3.70 | 4.05 | 3.35 |
| Tryptophan | 1.33 | 0.95 | 1.04 | 1.33 | 1.16 | 1.27 | 1.15 |
| Valine | 5.11 | 4.84 | 4.76 | 5.43 | 5.60 | 6.08 | 4.92 |
| Alanine | 4.29 | 4.84 | 4.64 | 4.98 | 5.81 | 4.30 | 3.66 |
| Asparagine acid | 11.45 | 11.47 | 10.67 | 7.86 | 6.97 | 19.62 | 5.24 |
| Glutamic acid | 18.92 | 17.79 | 18.10 | 19.16 | 21.12 | 18.73 | 27.12 |
| Glycine | 4.29 | 4.74 | 4.52 | 5.54 | 4.54 | 3.80 | 3.66 |
| Cystine | 1.53 | 1.79 | 1.39 | 1.66 | 1.37 | 1.77 | 2.20 |
| Proline | 5.32 | 4.63 | 4.87 | 6.87 | 10.03 | 0.00 | 12.46 |
| Serine | 5.11 | 5.68 | 4.99 | 4.76 | 4.75 | 4.30 | 4.61 |
| Tyrosine | 3.07 | 3.47 | 3.71 | 2.99 | 2.22 | 4.30 | 3.56 |
