# Supplementary figures and images for: The plant-based by-product diets for the mass-rearing of Acheta domesticus and Gryllus bimaculatus
Source: PLoS One. 2019 Jun 27;14(6):e0218830. doi: 10.1371/journal.pone.0218830 (PMC6597079; doi:10.1371/journal.pone.0218830)

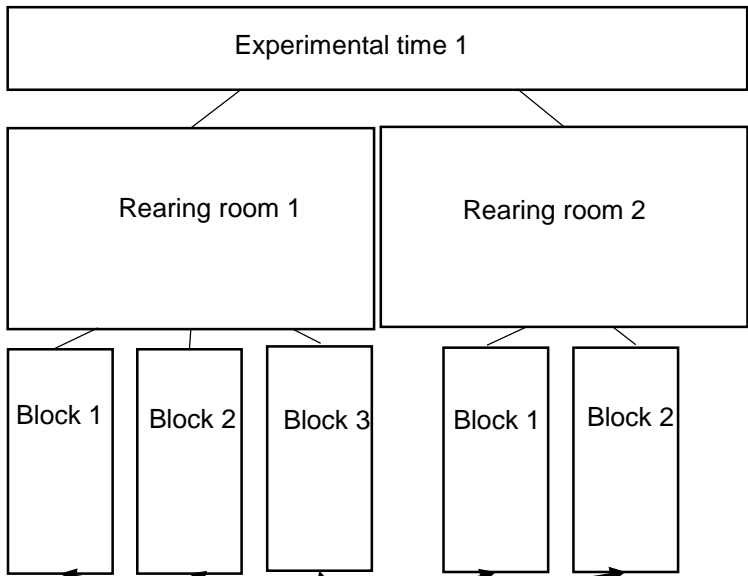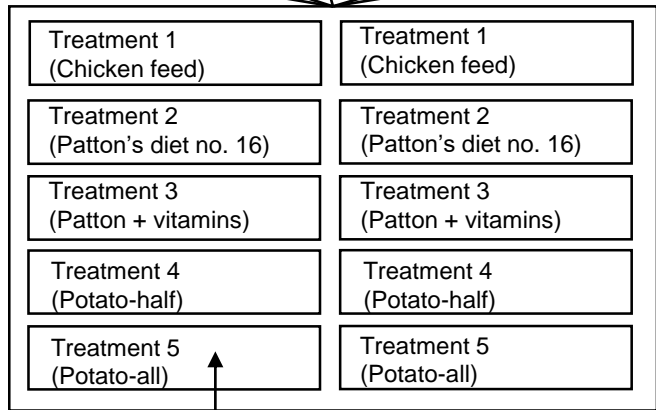

10 cricket individuals in each treatment replication

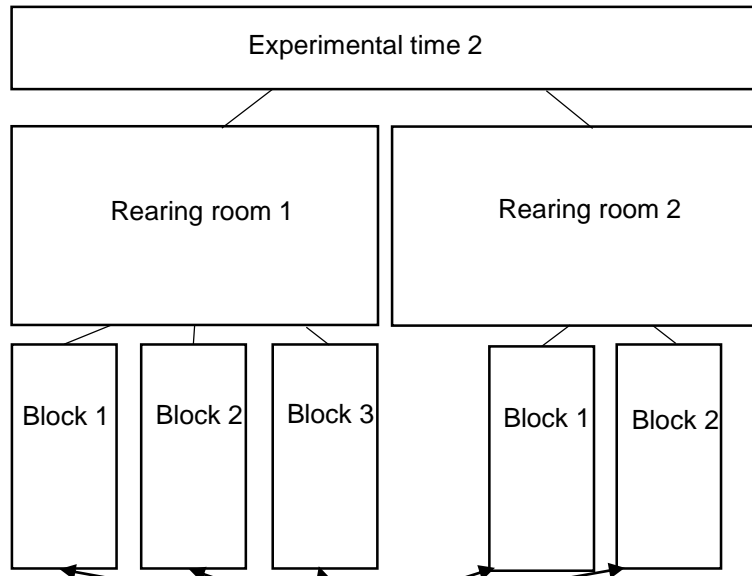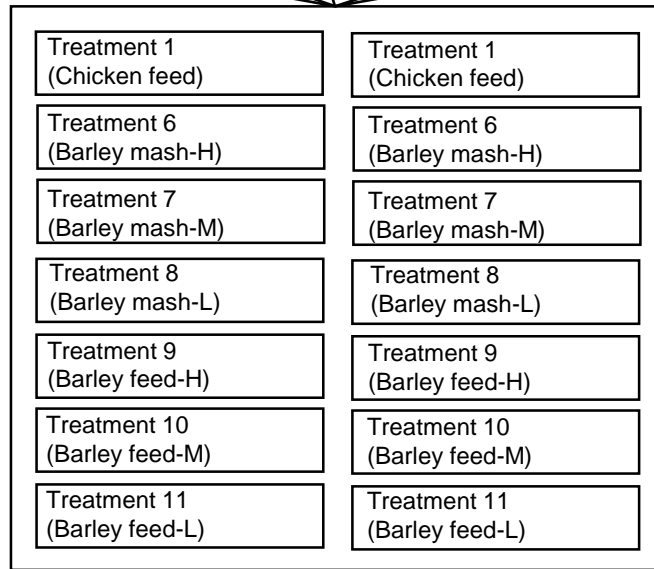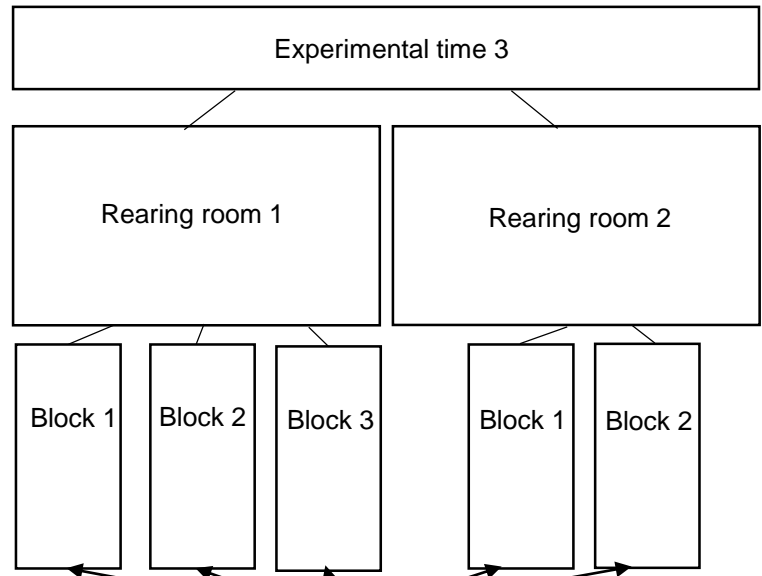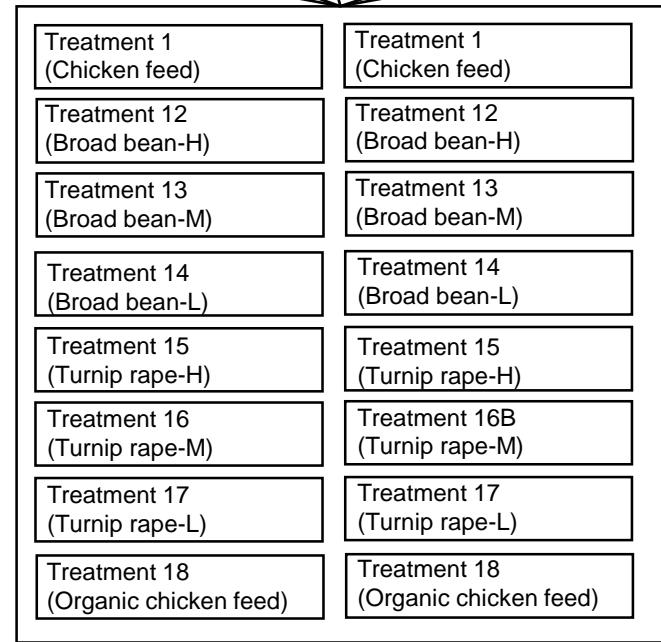

Supplement: S1 Fig — For both species, we conducted experiments in three different times (experimental time). In each time, the experiment was conducted in two thermally regulated rearing rooms, and five blocks were located in these rooms. The block was a growth chamber, where the temperature was microregulated with heat cables. There were five blocks in total for each species during each experimental time. Each block included two replicates of each diet treatment in separate containers, each container having one treatment. The control diet chicken feed was applied in each experimental time. Each container had ten cricket individuals. (PDF) [file pone.0218830.s006.pdf]

Percentage of amino acids

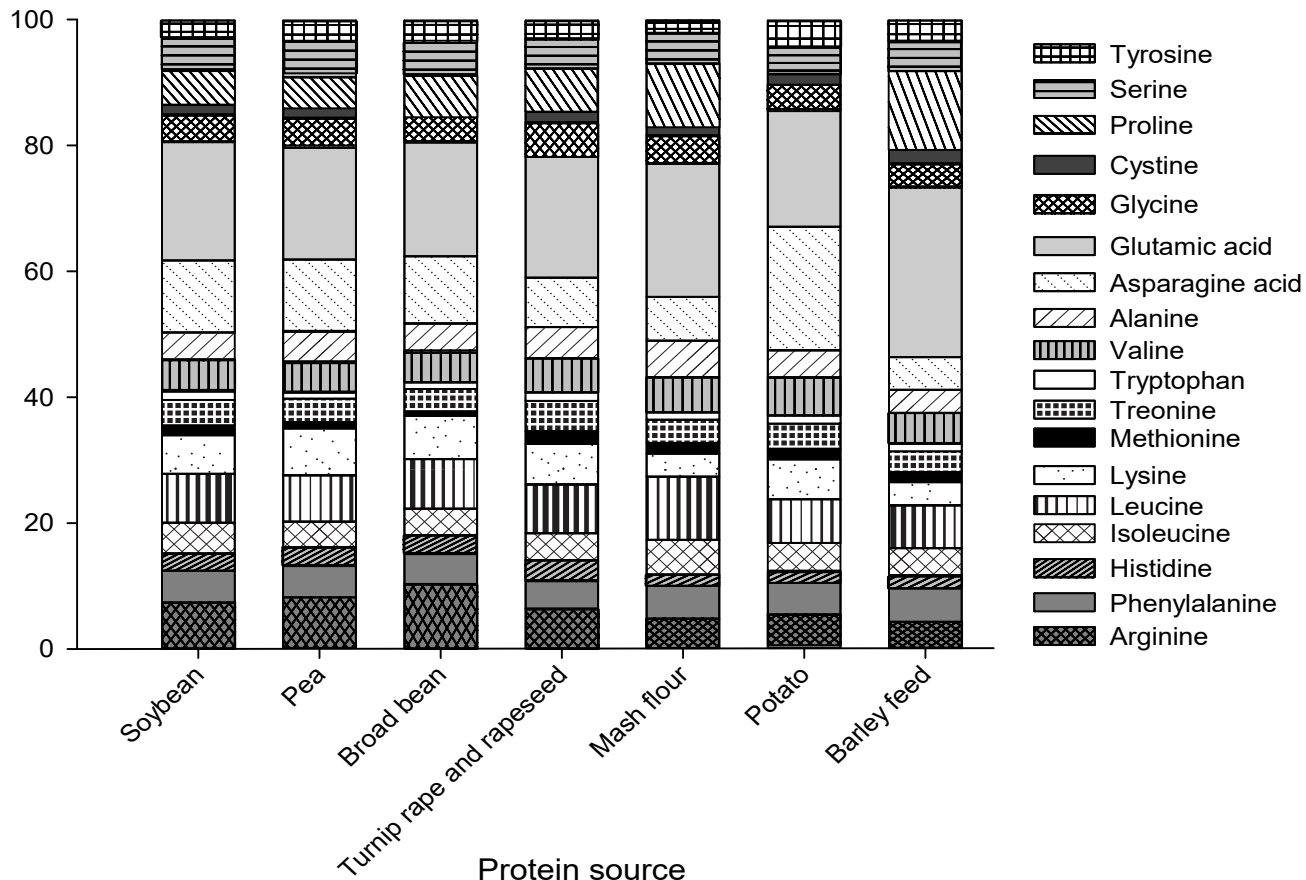

Supplement: S2 Fig — (TIF) [file pone.0218830.s007.tif]
